# Neuroprotective and cognitive enhancing effects of herbecetin against thioacetamide induced hepatic encephalopathy in rats via upregulation of AMPK and SIRT1 signaling pathways

**DOI:** 10.1038/s41598-024-61639-6

**Published:** 2024-05-18

**Authors:** Ahmed A. Sedik, Dalia T. Hussein, Khaled Fathy, Noha A. Mowaad

**Affiliations:** 1 Pharmacology Department, Medical Research and Clinical Studies Institute, National Research Centre, El-Buhouth St., Dokki, Cairo, 12622 Egypt; 2 Fellow of Biochemistry, Children Hospital, Faculty of Medicine, Mansoura University, Mansoura, Egypt; 3 Electron Microscopy Unit, Mansoura University, El Mansoura, 35516 Egypt; 4 Narcotics, Ergogenics and Poisons Department, Medical Research and Clinical Studies Institute, National Research Centre, El-Buhouth St., Dokki, Cairo, 12622 Egypt

**Keywords:** HE, Herbacetin, TAA, GS, SIRT1, AMPK, Annexin V, Biochemistry, Neuroscience

## Abstract

Acute liver injury, there is a risky neurological condition known as hepatic encephalopathy (HE). Herbacetin is a glycosylated flavonoid with many pharmacological characteristics. The purpose of this study was to assess the ability of herbacetin to protect against the cognitive deficits associated with thioacetamide (TAA) rat model and delineate the underlying behavioral and pharmacological mechanisms. Rats were pretreated with herbacetin (20 and 40 mg/kg) for 30days. On 30th day, the rats were injected with TAA (i.p. 350 mg/kg) in a single dose. In addition to a histpathological studies, ultra-structural architecture of the brain, liver functions, oxidative stress biomarkers, and behavioral tests were evaluated. Compared to the TAA-intoxicated group, herbacetin improved the locomotor and cognitive deficits, serum hepatotoxicity indices and ammonia levels. Herbacetin reduced brain levels of malodialdeyde, glutamine synthetase (GS), tumor necrosis factor- alpha (TNF-α), interleukin 1 B (IL-1β), annexin v, and increased brain GSH, Sirtuin 1 (SIRT1), and AMP-activated kinase (AMPK) expression levels. Also, herbacetin improve the histopathological changes and ultra- structure of brain tissue via attenuating the number of inflammatory and apoptotic cells. Herbacetin treatment significantly reduced the toxicity caused by TAA. These findings suggest that herbacetin might be taken into account as a possible neuroprotective and cognitive enhancing agent due to its ability to reduce oxidative stress, inflammation and apoptosis associated with TAA.

## Introduction

Acute liver failure (ALF) is an end result of hepatic injury and is characterized by the rapid advancement of hepatocellular damage and hepatic encephalopathy (HE). (HE) is a serious neuropsychiatric dysfunction that is clinically characterized by cognitive deficits and impaired or loss of consciousness levels^1^. Due to its hepatotoxic and neurotoxic effects, thioacetamide (TAA) has been used frequently to induce (HE)^2^.The pathophysiology of HE is distinct and complicated, and it is still unclear what causes the insults to the hepatic and neural systems in that order However, it is generally accepted that hyper ammonia, oxidative stress and neuro inflammation which damages cellular DNA and enzymes are the possible reasons for the clinical, pathological, and neurochemical changes in HE^3^.

Numerous investigations have been conducted to create safe alternative medications especially from natural sources, hence herbacetin (3,4′,5,7,8-pentahydroxyflavone), a novel bioactive glycosylated flavonoid that is frequently distributed in medicinal plants in various families and genera, including Drosera, Potentilla, Orostachys, Melaleuca, Nerium, Zanthoxylum, and Buddleja^4^. Herbacetin has been reported to exert a wide range of pharmacological effects; including; antioxidant, hepato-protective, anti-obesity, anti-hyperglycemic, anti-cancer, anti-viral, antibacterial, and anti-inflammatory characteristics^5^. Additionally, studies have demonstrated that herbacetin has potent anti-inflammatory effects by preventing the release of pro inflammatory cytokines including tumor necrosis factor alpha (TNF-α) and interlukin- 1beta (IL-1β) and reducing the synthesis of cellular nitric oxide (NO)^6^.

SIRT1, a nicotinamide adenine dinucleotide (NAD +)-dependent deacetylase with crucial regulatory roles in numerous physiological processes. It is the mammalian homolog of the yeast Sir2 protein including, seven members (SIRT 1–SIRT^7^). Sirt1 specifically is thought to be a potential target for neurodegenerative diseases due to its neuroprotective properties and generally extends the survival and lifespan of mammalian cells. Amplification of SIRT1 function has been proposed as a suitable approach to treat these disorders because it has been shown to be a contributing factor in liver diseases, particularly non-alcoholic fatty liver disease, inflammation and oxidative stress are frequent symptoms of many liver illnesses^8^.

SIRT1's catalytic activity is regulated by many molecular pathways^9^. The level of NAD + detects the activity of SIRT1 substrate to control a cell's energy status. When cells experience a lack of energy, the AMP/ATP ratio rises, and AMP-activated kinase (AMPK), is subsequently phosphorylated and activated^10,11^. AMPK is a serine/threonine protein kinase complex consisting of a catalytic α-subunit (α1 and α2), a scaffolding β-subunit (β1 and β2) and a regulatory γ-subunit (γ1, γ2 and γ3). AMPK has been considered to be an important therapeutic target for controlling many human diseases. Together, SIRT1 and AMPK stimulate each other in a bi-directional pathway^12^.

To the best of our knowledge, just a few research projects have been carried out to investigate the biological functions of herbacetin. Therefore, the purpose of the current work was to determine whether herbacetin can reduce neurocognitive deficits associated with HE in rats.

## Materials and methods

### Chemicals

Herbacetin was obtained from Merck (CAS NO: 527-95-7), Germany. Thioacetamide (TAA) was purchased from Sigma-Aldrich, USA (CAS NO: 62-55-5). Herbacetin and TAA were freshly prepared in saline.

### Animals

Adult male Sprague dawly rats weighing 150–175 gm. were obtained from the animal breeding unit, National Research Centre (NRC, Giza, Egypt). Rats were acclimatized for one week before carrying out any experiments and were kept under standard environmental conditions (temperature, 25 °C, humidity, 50%, light system 12:12 dark/light system). A commercial diet and water were provided ad libitum*.* The experimental study was carried out following the relevant guidelines and regulations approved by the Medical Research Ethics Committee (MREC) of the National Research Centre (approval number; 01451023). The study was reported in accordance with ARRIVE guidelines.

### Experimental design

Thirty-two male Sprague dawly rats were randomly allocated into four groups (8 rats each) as followed: Group I: Rats were given saline orally as control group for 30 days. Group II: Rats were injected with TAA (350 mg/kg. b.w, i.p.) on the 30th day of the experiment, and acted as the model group^13^, Group III and IV: rats were orally pretreated with herbacetin (20 and 40 mg/kg b.w, respectively) for 30 days and were given TAA (350 mg/kg b.w) on the 30th day of the experiment^14^. After TAA injection, supportive therapy (consisted of 10% glucose mixed with lactate ringer (1 v:1 v) was given subcutaneously to prevent hypoglycemia and renal failure^15^.

**Figure 1:**
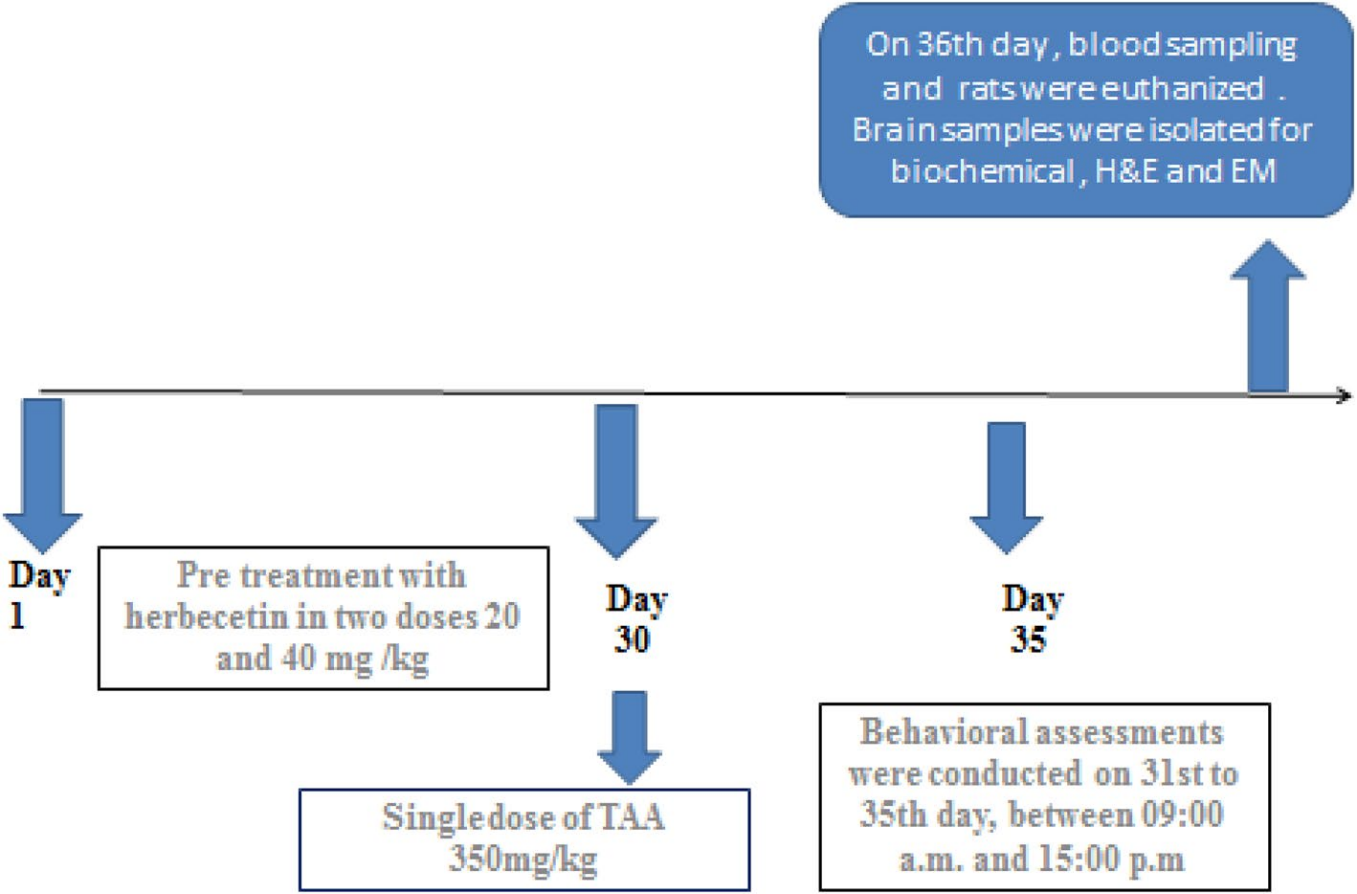
The timeline and experimental design. Pretreatment of rats with herbecetin started on the 1st day to 30th day. (TAA administration on 30th day). Behavioral assessment on 31st to 35th day.

Behavioral assessments were conducted on 31st to 35th day, between 09:00 a.m. and 15:00 p.m. Twenty-four h following the behavioral tests; blood samples were collected from the retro-orbital plexus, left to clot, and centrifuged at 4000 rpm for 10 min for serum separation.On 36th day ,, rats were euthanized and brain samples were excised as illustrated in (Fig. 1). Brain samples were stored at − 80 °C for the subsequent gene expression and biochemical analyses. Moreover brain specimens were fixed in 10% buffered formalin and 4% glutraldyde for histopathological and ultra- structural studies, respectively.

### Behavioral tests

#### Rotarod test

A rotarod device (Model No. 7750; Ugo Basile) was employed to assess the motor coordination of the rats. They underwent three training sessions consecutively over three days on the rotarod, set at a constant speed of 4 rotations per minute (rpm), until achieving consistent performance. The initial falling latency time was recorded for rats trained on the rotarod, and the subsequent falling latency time was measured post-treatment^16^.

#### Novel object recognition test (NOR)

Before conducting the actual test, the rats were given three days, with each session lasting two minutes, to freely explore the apparatus. This period of exploration allows the rats to become familiar with their surroundings and possibly the tasks they will need to perform during the test. It helps reduce the likelihood of their behavior being influenced by novelty or fear of the environment during the actual experiment. On the testing day, two trials were conducted: in the first trial (T1), two identical objects (F) were positioned in two opposite corners of the apparatus. In the subsequent trial (T2), one of the identical objects from the first trial (T1) was replaced with a novel object (N). Rats were then exposed to two distinct objects: the familiar (F) and the novel (N). The discrimination index (DI) was computed as the difference in exploration time, expressed as a percentage of the total time spent examining both items in T2. The formula used to calculate DI was DI = (N–F)/(N + F)^17^.

#### Y maze

The Y-maze test was utilized to evaluate spatial working memory in rats with HE induction. In brief, rats were positioned in the center of a wooden Y-maze apparatus, which was labeled A, B, and C, with arms set at 120 degrees, 40 cm in length, and 35 cm in height, for a duration of 5 min. The number of entries into each arm, along with the spontaneous alternation behavior, were examined to gauge motor activity and the rats' inherent propensity to alternate between the three arms, respectively. The spontaneous alternation percentage (SAP) was determined using the following formula: SAP = (Total alternations/(Number of entries–2)) × 100^18^.

### Biochemical assessment

#### Determination of hepatic toxicity indices and ammonia levels

Serum levels of alanine aminotransferase (ALT), aspartate aminotransferase (AST), and alkaline phosphatase (ALP) were assessed using commercially available kits obtained from Bio diagnostics, located in Cairo, Egypt. Additionally, ammonia levels were determined using kits obtained from the same source*.*

#### Preparation of brain homogenate and estimation of oxidative stress biomarkers and inflammatory mediators

The rats were euthanized by decapitation under light anesthesia immediately after blood sampling, Blood was drawn from the eye’s retro-orbital plexus of the rats after being anesthetized with ketamine (100 mg/kg) and xylazine (10 mg/kg). And their brain tissues were swiftly excised and stored at – 80 °C for subsequent biochemical analysis. The brain tissues were homogenized in ice-cold PBS to create a 20% w/v homogenate for the measurement of brain ammonia levels^19^. Moreover, brain homogenate was utilized for estimating the brain contents of malondialdehyde (MDA), reduced glutathione (GSH), glutamine synthetase (GS), tumor necrosis factor-alpha (TNF-α), and interleukin-1beta (IL-1β). MDA levels were determined using the method described by Ref.^20^. GSH levels were measured following the method outlined in reference^21^. The tissue levels of (GS) were assessed via (an enzyme-linked immunosorbant assay) ELISA using a test reagent kit from MyBioSources. The brain level of tumor necrosis factor-alpha (TNF-α) was determined using (ELISA) and a test reagent kit from Raybiotech, according to the method described by Ref.^22^. The tissue levels of interleukin-1beta (IL-1β) were also determined via ELISA using a test reagent kit from ImmunoBiological Laboratories, in accordance with the method detailed in reference^23^.

### Real-time polymerase chain reaction (PCR) quantification of SIRT1 and AMPK

The mRNA expression levels of SIRT1 and AMPK genes were evaluated using real-time PCR, which was standardized by co-amplification with the housekeeping GAPDH gene serving as an internal control. RNA extraction from brain tissue was conducted using Trizol reagent. The RNA was reverse-transcribed using M-MLV reverse transcriptase (Invitrogen, Carlsbad, CA, USA) and then utilized for PCR with specific primers. Quantification of SIRT1 and AMPK was performed using SIRT1 and AMPK RT-PCR fluorescence diagnostic kits in accordance with the manufacturers' instructions. For amplification, 40 cycles were run, consisting of 95 °C for 30 s, 56 °C for 1 s, and 72 °C for 50 s, followed by 1 min at 60 °C and 1 min at 72 °C.

SIRT1 forward primer 5′—TGC CAT CAT GAA GCC AGA GA-3′, SIRT1 reverse primer 5′—CAT CGC AGT CTC CAA GAA GC -3′

AMPK forward primer 5′—CACCCTGAAAGAGTACCGT-3′, AMPK reverse primer 5′—CATTTTGCCTTCCGTACACCT-3′, using Rotor-Gene Q5 plex real-time Rotary analyzer (Corbettlife sciences, USA).

### Flow cytometry analysis for apoptosis assessment

The brain levels of annexin V (AnnV) were assessed using propidium iodide (PI) staining and flow cytometry, following the provided instructions. This method allows for the determination of the number of necrotic and apoptotic cells in the brain tissue^24^.

### Histopathological examination of the hippocampus

The hippocampus was swiftly dissected, fixed in 10% neutral buffered formalin, and subsequently embedded in paraffin wax. Sections of 4 μm thickness were prepared and stained with Hematoxylin and Eosin (H&E). These stained sections were then examined using a binocular Olympus CX31 microscope^25^.

### Electron microscopic examination of the hippocampus

The hippocampus specimen was prepared for examination using an electron microscope by immersing it in 2.5% glutaraldehyde in phosphate buffer. Subsequently, the specimen was washed with phosphate buffer and fixed in 1% osmium tetroxide. It was then dehydrated using ascending ethanol concentrations (100%, 95%, and 70%) and embedded in epoxy resin capsules.

To localize the selected area, the specimen was stained with toluidine blue and examined under a light microscope. Ultrathin sections were then cut using a diamond knife on copper grids and stained with uranyl acetate followed by lead citrate staining. Finally, the grids were examined and photographed using a JEOL-JEM-100 SX electron microscope at 80 kilovolts at the electron microscope unit of the Faculty of Agriculture, Mansoura University, Japan^26^.

### Statistical analysis

The results were presented as the mean ± SD (n = 8). Significant differences among groups were assessed using One-way ANOVA with LSD post hoc tests, followed by the Duncan test for all comparisons. Before conducting ANOVA, all samples were evaluated for normality using the Shapiro–Wilk test. Data analysis was performed using GraphPad Prism v. 8.0 (GraphPad Software, Inc., CA, USA). Differences were considered significant when the p-value was ≤ 0.05.

### Ethics approval

In accordance with the National Regulations for Animal Welfare, experiments were carried out. Following the approval of the animal experimental protocol by the "NRC's Committee on Animal Care and Use's ethics committees" (approval number: MREC-01451023), clearance was given by the Institutional Animal Ethical Committee (IAEC).

## Results

### Impact of herbacetin administration on falling off time in rotarod test, and DI % in rats intoxicated with TAA

**Figure 2:**
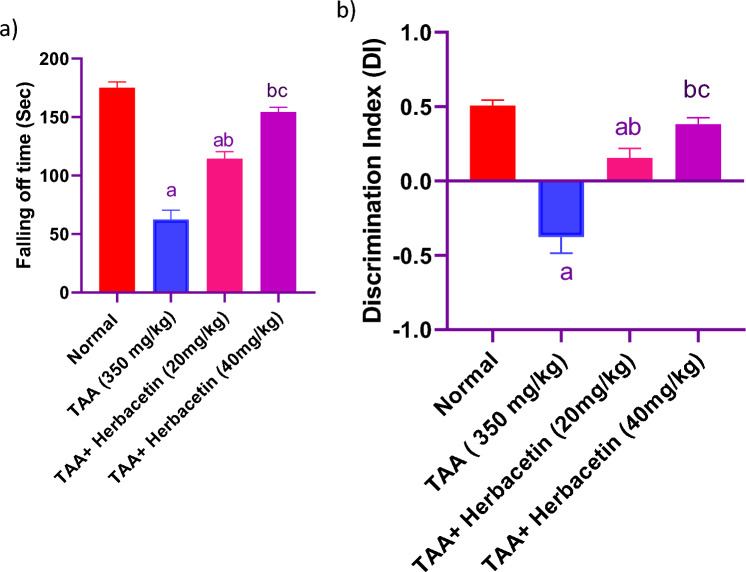
Impact of herbacetin administration on (**a**) motor coordination, and (**b**) discrimination index (DI %) in rats intoxicated with (thioacetamide (TAA)). Rats were pretreated with herbacetin (20 and 40 mg/kg) for 29 days and administrated TAA (i.p. 350 mg/kg) in a single dose. Motor coordination and discrimination index were evaluated. Results are expressed as mean ± standared deviation (SD) (n = 8). ^a^Significant difference from normal group *P* < *0.05*. ^b^Significant difference from TAA group *P* < *0.05*. ^c^Significant difference from TAA + Herbacetin (20 mg/kg) group P < 0.05.

Rats received TAA exhibited a decrease in falling off time in rotarod test about 35% of normal values. Oral administration of Herbacetin at 20 and 40 mg/kg to rats intoxicated with TAA could increase it nearly to 1.83 fold, and 2.5 fold, respectively, as compared with TAA group. Regarding, NOR test, TAA-treated rats showed an inability to distinguish between familiar and novel items. The novel item was considerably distinguished from the familiar object in rats pre- treated with Herbacetin at 20 and 40 mg/kg in conjunction with TAA. Herbacetin 40 mg/kg nearly normalized all the behavioral tests (Fig. 2).

### Impact of herbacetin administration on Y maze indices in rats intoxicated with TAA

**Figure 3:**
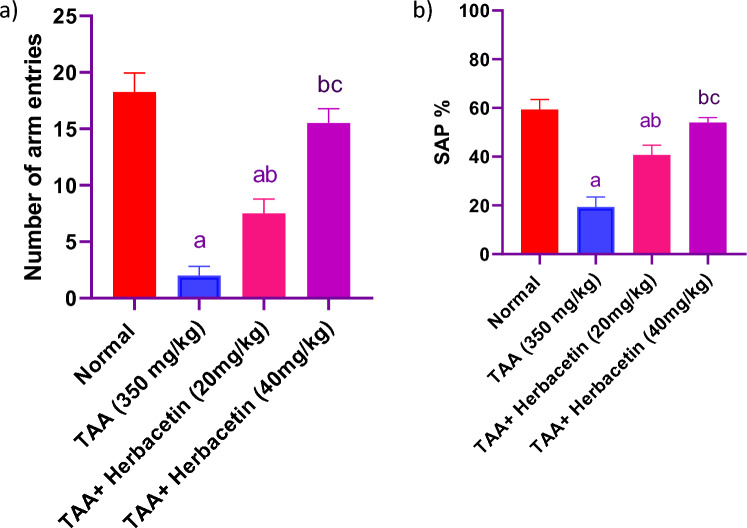
Impact of herbacetin administration on Y maze (**a**) number of arm entries, and (**b**) spontaneous alternation percentage (SAP %) in rats intoxicated with TAA. Rats were pretreated with herbacetin (20 and 40 mg/kg) for 29 days and administrated thioacetamide (TAA) (intraperitoneal (i.p), 350 mg/kg) in a single dose. Y maze indices were evaluated. Results are expressed as mean ± standared deviation (SD) (n = 8). ^a^Significant difference from normal group *P* < *0.05*. ^b^Significant difference from TAA group *P* < *0.05*. ^c^Significant difference from TAA + Herbacetin (20 mg/kg) group *P* < *0.05*.

TAA-intoxicated rats significantly reduced the alternations when compared to normal rats. Meanwhile, pretreatment with Herbacetin, 20 mg/kg and 40 mg/kg markedly elevated the frequencies of entering the maze arms. Interestingly, pretreatment with Herbacetin at a dose of 40 mg/kg markedly elevated the spontaneous alternations as compared to TAA-intoxicated group (Fig. 3).

### Impact of herbacetin administration on the hepatic and brain ammonia levels in rats intoxicated with TAA

**Figure 4:**
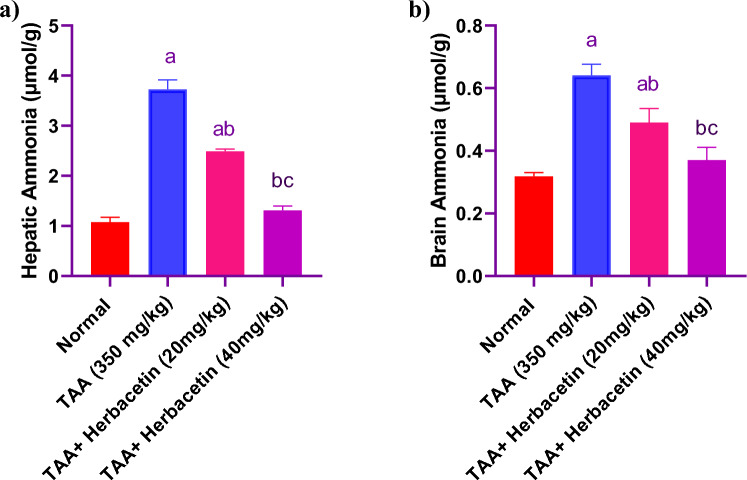
Impact of herbacetin administration onthe hepatic and brain ammonia levelsin rats intoxicated with TAA. Rats were pretreated with herbacetin (20 and 40 mg/kg) for 29 days and administrated thioacetamide (TAA) (intraperitoneal (i.p), 350 mg/kg) in a single dose. Hepatic and brain ammonia levels were evaluated. Results are expressed as mean ± standared deviation (SD) (n = 8). ^a^Significant difference from normal group *P* < *0.05*. ^b^Significant difference from TAA group *P* < *0.05*. ^c^Significant difference from TAA + Herbacetin (20 mg/kg) group *P* < *0.05*.

Administration of TAA revealed a marked elevation in the hepatic and brain ammonia nearly to 3.5 fold and twofold, respectively, when compared to normal control rats. Hepatic and brain ammonia levels were significantly decreased in (TAA + Herbacetin -20 group) by 66% and 76%, respectively, as compared with TAA group. While rats in (TAA + Herbacetin -40 group) restored the normal levels of hepatic and brain ammonia indices (Fig. 4).

### Impact of herbacetin administration on ALT, AST, and ALP levels in rats intoxicated with TAA

**Figure 5:**
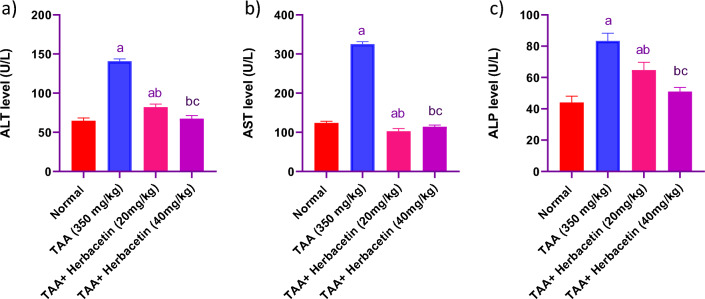
Impact of herbacetin administration on (**a**) ALT, (**b**) AST, and (**c**) ALP levels in rats intoxicated with TAA. Rats were pretreated with herbacetin (20 and 40 mg/kg) for 29 days and administrated TAA (intraperitoneal (i.p), 350 mg/kg) in a single dose. Alanine aminotransferase (ALT), aspartate aminotransferase (AST), and alkaline phosphatase (ALP) were evaluated. Results are expressed as mean ± standared deviation (SD) (n = 8). ^a^Significant difference from normal group *P* < *0.05*. ^b^Significant difference from TAA group *P* < *0.05*. ^c^Significant difference from TAA + Herbacetin (20 mg/kg) group *P* < *0.05*.

Administration of TAA resulted in a prominent elevation in the serum levels of ALT, AST, and ALP nearly to 2.18 fold, 2.62 fold and 1.89 fold, respectively, when compared to normal rats. ALT, AST, and ALP levels were significantly decreased in (TAA + Herbacetin -20 group) by 57%, 32% and 77%, respectively, as compared with TAA group. While pretreatment with (TAA + Herbacetin -40) restored the normal levelsof hepatotoxicity indices (Fig. 5).

### Impact of herbacetin administration on the brain levels of GS, MDA, and GSH in rats intoxicated with TAA

**Figure 6:**
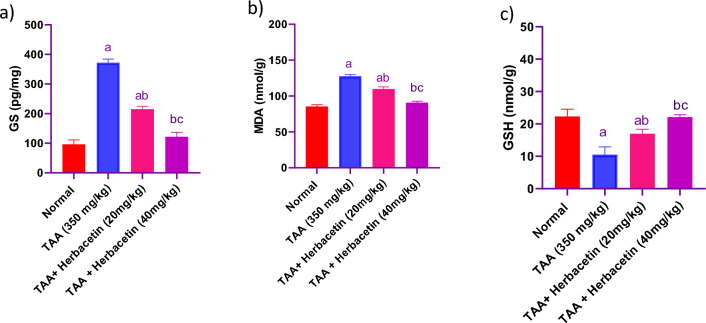
Impact of herbacetin administration on (**a**) GS, (**b**) MDA, and (**c**) GSH levels in rats intoxicated with TAA. Rats were pretreated with herbacetin (20 and 40 mg/kg) for 29 days and administrated thioacetamide (TAA) intaperitoneal (i.p), 350 mg/kg) in a single dose. Expression brain levels of glutamine synthetase (GS), malondialdeyde (MDA) and reduced glutathione (GSH) were evaluated. Results are expressed as mean ± standared deviation (SD) (n = 8). ^a^Significant difference from normal group *P* < *0.05*. ^b^Significant difference from TAA group *P* < *0.05*. ^c^Significant difference from TAA + Herbacetin (20 mg/kg) group *P* < *0.05*.

Rats received TAA (350 mg/kg, i.p.) were associated with a rise in brain GS and MDA levels of about 3.8 fold and 1.5 fold of normal values, respectively and a decrease in brain GSH levels of about 47% of normal values. While rats in group (TAA + Herbacetin -20 were associated with a decrease in brain GS and MDA levels of about 57% and 86 fold of TAA values, respectively and an increase in brain GSH levels of about 161% of TAA values. While rats in group (TAA + Herbacetin -40) were associated with a normalized values of GS, MDA, and GSH (Fig. 6).

### Impact of herbacetin administration on the brain levels of TNF-α, and IL-1β in rats intoxicated with TAA

**Figure 7:**
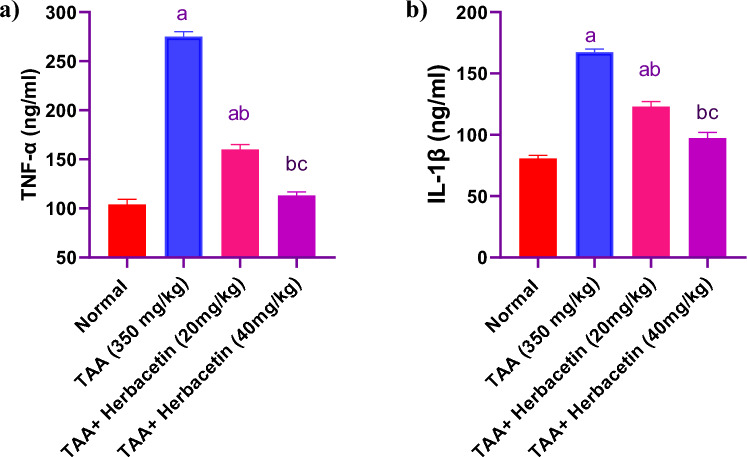
Impact of herbacetin administration on (**a**) TNF-α, and (**b**) IL-1β levels in rats intoxicated with TAA. Rats were pretreated with herbacetin (20 and 40 mg/kg) for 29 days and administrated thioacetamide (TAA) intraperitoneal (i.p), (350 mg/kg) in a single dose.Expression brain levels of alpha tumor necrotic factor (TNF-α) and (**b**) interlukin 1 B (IL-1β) were evaluated. Results are expressed as mean ± standared deviation (SD) (n = 8). ^a^Significant difference from normal group *P* < *0.05*. ^b^Significant difference from TAA group *P* < *0.05*. ^c^Significant difference from TAA + Herbacetin (20 mg/kg) group *P* < *0.05*.

Intra-peritoneal injections of TAA at 350 mg/kg was associated with a significant increase in brain TNF-α, and IL-1β levels of about 2.6 fold and twofold of normal values, respectively. Levels of TNF-α and IL-1β were significantly decreased in TAA-rats pretreated with Herbacetin -20 by 58% and 73%, as compared with TAA group. Pretreatment with Herbacetin -20 restored the normal levels of brain TNF-α, and IL-1β levels (Fig. 7).

### Impact of herbacetin administration on the relative brain expression of SIRT1 and AMPK in rats intoxicated with TAA

**Figure 8:**
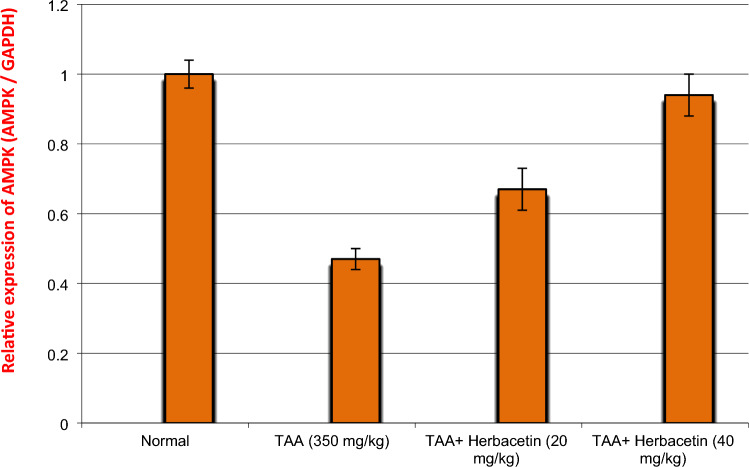
Impact of herbacetin administration on the relative brain expression of AMPK in rats intoxicated with TAA. Rats were pretreated with herbacetin (20 and 40 mg/kg) for 29 days and administrated thioacetamide (TAA) (intraperitoneal (i.p), (350 mg/kg) in a single dose. Expression brain levels of AMP-activated kinase (AMPK) were evaluated. Results are expressed as mean ± standared deviation SD (n = 8). ^a^Significant difference from normal control group *P* < *0.05*. ^b^Significant difference from TAA group *P* < *0.05*. ^c^Significant difference from TAA + Herbacetin (20 mg/kg) group *P* < *0.05*.

**Figure 9:**
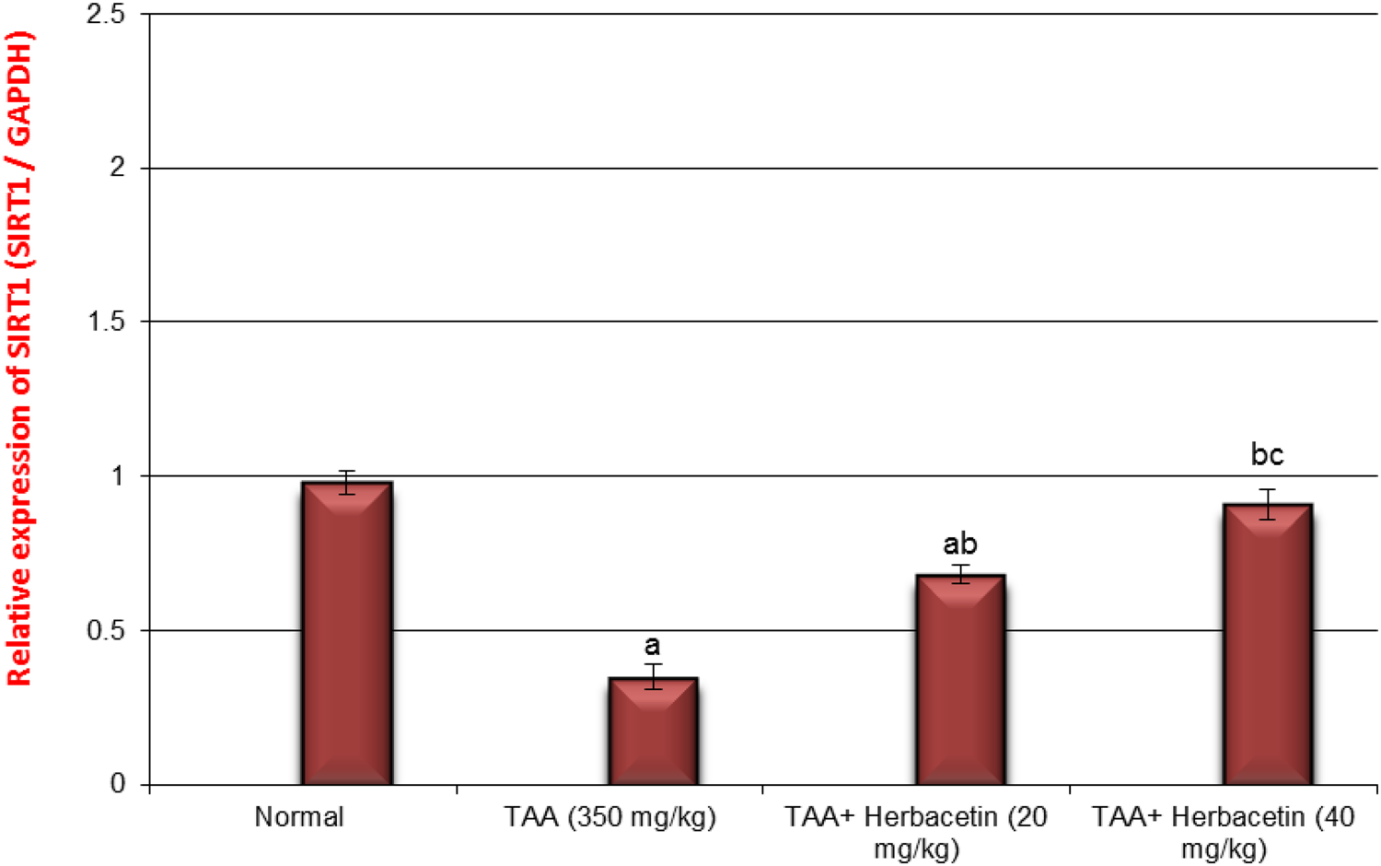
Impact of herbacetin administration on the relative brain expression of SIRT1 in rats intoxicated with TAA. Rats were pretreated with herbacetin (20 and 40 mg/kg) for 29 days and administrated thioacetamide (TAA) intaperitoneal (i.p), (350 mg/kg) in a single dose. Expression brain levels of Siritin 1 (SIRT1) were evaluated. Results are expressed as mean ± standared deviation (SD) (n = 8). ^a^Significant difference from normal group P < 0.05. ^b^Significant difference from TAA group P < 0.05. ^c^Significant difference from TAA + Herbacetin (20 mg/kg) group P < 0.05.

The expression levels of SIRT1 and AMPK genes in brain tissues are shown in Figs. 7, 8. The expression levels of SIRT1 and AMPK genes in rats intoxicated with TAA were down regulated significantly by 44%, and 23%, respectively, as compared to those in control normal rats. However, the expression levels of SIRT1 and AMPK genes in TAA-rats treated with Herbacetin -20 were increased significantly by 1.8 fold and 2.8 fold, respectively, compared to those in TAA rats. Moreover, the expression levels of SIRT1 and AMPK genes in TAA-rats treated with Herbacetin -40 were elevated significantly by 2.6 fold and, 4.6 fold%, respectively, compared to those in TAA rats (Figs. 8, 9).

### Impact of herbacetin administration on annexin V expression in brain tissue in rats intoxicated with TAA

**Figure 10:**
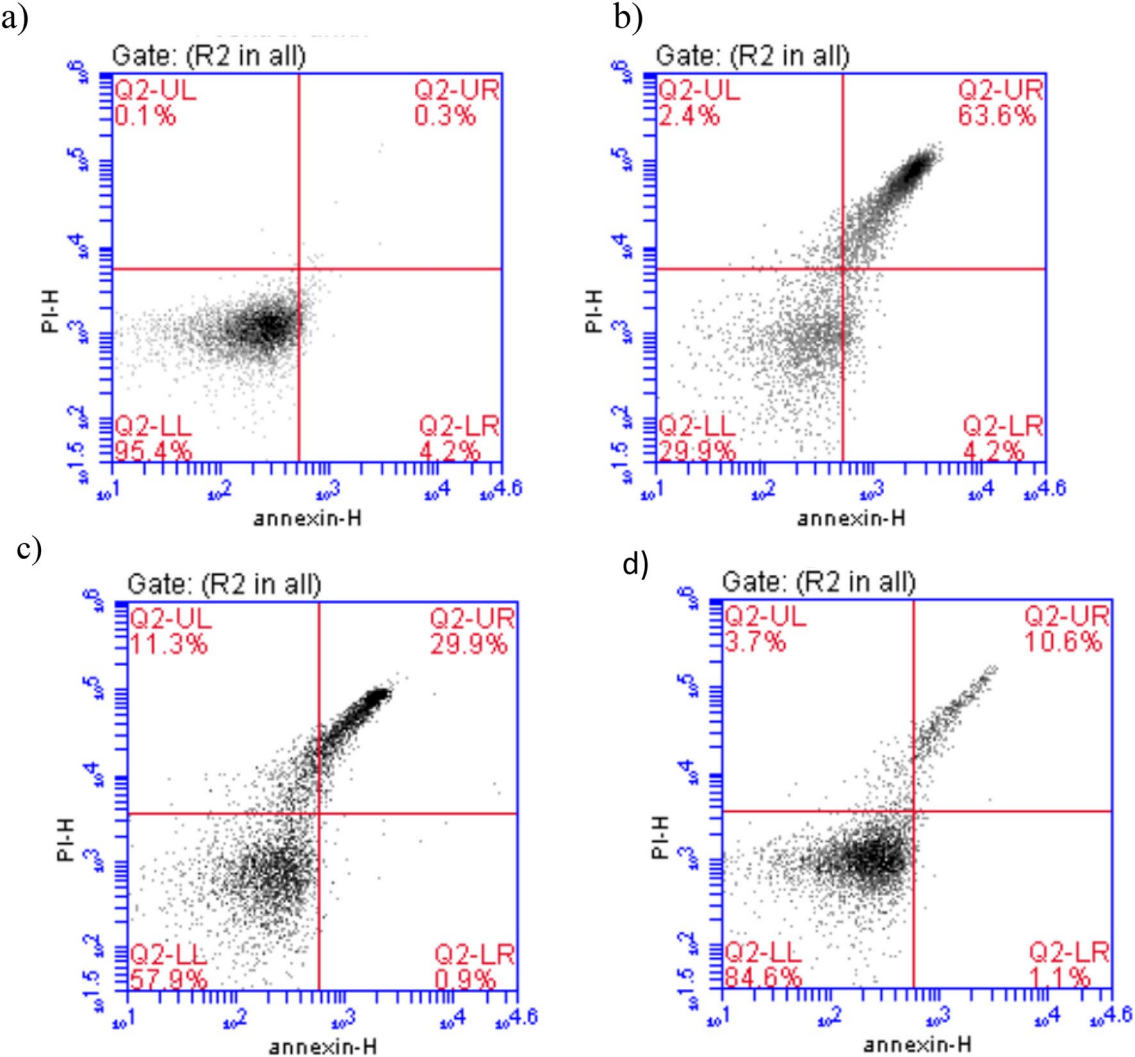
Impact of herbacetin administration on annexin V expression in brain tissue in rats intoxicated with TAA. Rats were pretreated with herbacetin (20 and 40 mg/kg) for 29 days and administrated thioacetamide (TAA) intraperitoneal (i.p), (350 mg/kg) in a single dose. Expression brain levels of annexin V were evaluated. Flow cytometry histogram of TAA (350mg/kg) group, showing higher levels of annexin V (63.6%) than in normal control (0.3%). While herbacetin (20 and 40 mg/kg) showing lower levels of (29.9% and 10.6%) respectively, than in TAA (350mg/kg) group. (**a**) Normal (**b**) TAA(350mg/kg) (**c**) TAA+ Herbacetin(20mg/kg) (**d**) TAA+ Herbacetin(40mg/kg).

The expression levels of annexin V were evaluated by flow cytometry in the brain homogenate from different experimental groups. We observed a significant increase (p < 0.05) in annexin V expression to 63.6% in TAA group, as compared with the normal group. However, administration of Herbacetin at 20 and 40 mg/kg showed a significant decrease (p < 0.05) in annexin V expression to 29.9 and 10.6%, respectively, compared with TAA group (Fig. 10).

### Impact of herbacetin administration on the histopathological pictures of the hippocampus of rats intoxicated with TAA

**Figure 11:**
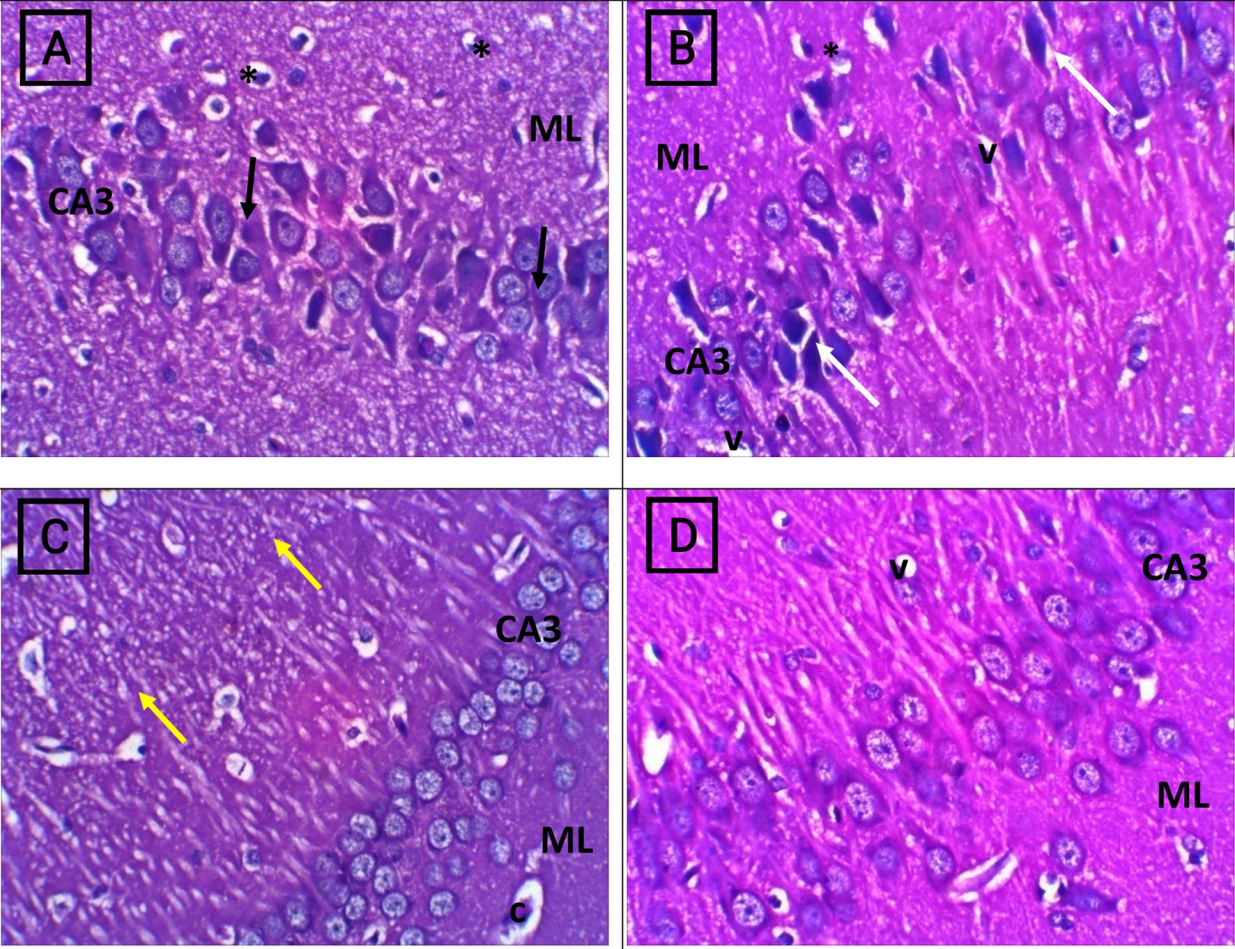
Photomicrographs of H&E staining rat brain (hippocampus) sections of control and different experimental groups, (**A**) Normal group Shows three normal layers of large pyramidal cells in Cornu Ammonis region (CA3), also with vesicular nuclei (arrows). Molecular layer (ML) shows many glial cells (*) among neuronal processes. (**B**) thioacetamide (TAA) group showing marked disorganization, marked apoptosis of large pyramidal cells (white arrow), appear of few vacuoles (V) in Molecular layer and decreased population of granular cells and necrosis. (**C**) TAA + Herbacetin 20 mg/kg group showing mild histological appearance of CA3, few shrunken neurons with faded nucleus (yellow arrow).Molecular layer (ML) mostly shows normal. Molecular layer shows normal, Widened capillaries (c). (**D**) TAA + Herbacetin 40 mg/kg showing preservation of small pyramidal cells with no change in their size in CA3 that gaining its layers, with mild neuropil vacuolation (v). Molecular layer (ML) mostly shows normal size of cells & fibers. Magnification X: 400.

Photomicrographs sections from the hippocampus of normal rats exhibited normal architecture. While, rats intoxicated with TAA (350mg/kg) group showed significant presence of necrosis and apoptosis in pyramidal cells with appearance of few vacuoles in molecular layer and decreased population of granular cells. On the other hand, rats received TAA + Herbacetin 20mg/kg group revealed mild histological alterations, evidenced by few shrunken neurons with faded nucleus and slight changes in molecular layer. Rats received TAA + Herbacetin 40 mg/kg exhibited mild vacuolation with normalized appearance of molecular layer (Fig. 11).

### Impact of herbacetin administration on the ultra- structural pictures of the hippocampus of rats intoxicated with TAA

**Figure 12:**
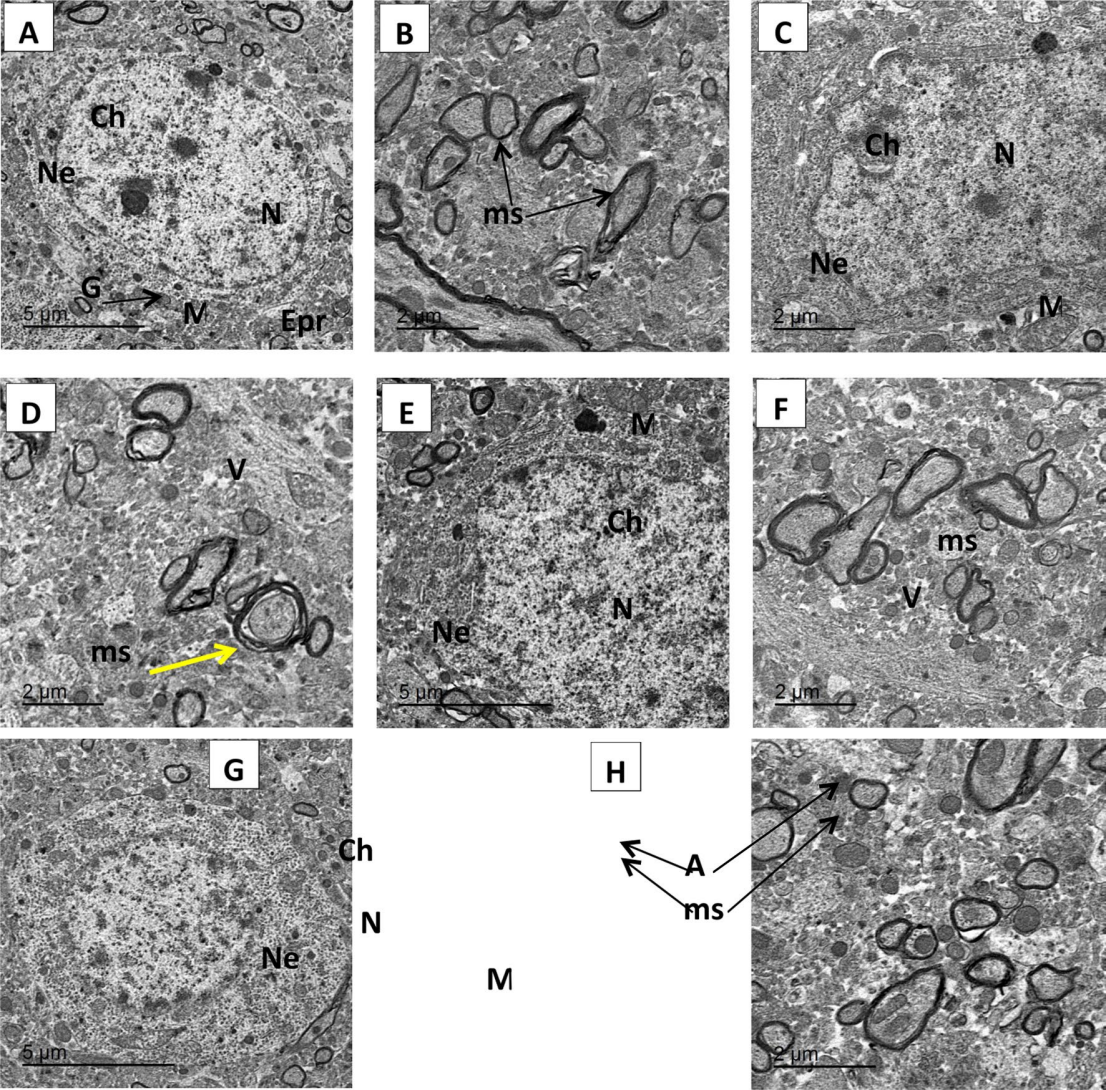
Electron micrographs of rat hippocampus of control and different experimental groups: (**A**,**B**) of normal group where (**A**) showing neurocytes with normal nucleus (N) with an intact nuclear membrane (Ne) and smooth chromatin (Ch) Normal mitochondria (M), normal endoplasmic reticulum (Epr) and Golgi bodies (G) were widely distributed in the cytoplasm.of neurons in the hippocampus, with intact mitochondrial structures and obvious mitochondrial cristae. (**B**) Showing normal myelinated axons exhibited dark ring-shaped myelin sheaths (ms) surrounding the axon. (**C**,**D**) thioacetamide (TAA group) (350 mg/kg) Severe pathological changes characterized by damage of neuronal nuclei losing its round shape, irregular and wide nuclear membrane (Ne) and progressive condensation of chromatin (Ch) also fig (**D**) showing myelin breakdown or disruption as disjointed and unravelling myelin layers (yellow arrow) and spreading of vacuoles (V) indicating neuronal apoptosis. (**E**,**F**) TAA + Herbacetin 20 mg/kg group showing neurnal nuclei retained its normal shape with small projection of nuclear membrane and slight aggregation of chromatin (Ch) and still widen of some mitochondria (M). Fig (**F**) showing small amount vacuoles are observed. (**G**,**H**) TAA + Herbacetin 40 mg/kg group showing noticed development neurnocyte comparing with normal group, where nuclei retained its normal shape , surrounded by normal distribution of normal mitochondria (M) and fig (H) showing Normal axon (A) in cross-section surrounded by thick and tight myelin sheath MS.

The hippocampus of normal rats revealed neurocytes with normal nucleus (N) with an intact nuclear membrane. Meanwhile, hippocampus of rats that received TAA showed an obvious damage of neuronal nuclei, irregular and wide nuclear membrane and neuronal apoptosis. Conversely, the hippocampus of rats that received TAA + Herbacetin 20mg/kg group retained its normal shape with small projection of nuclear membrane and slight aggregation of chromatin and still widen of some mitochondria with small amount vacuoles., rats received TAA + Herbacetin 40 mg/kg succeeded to restore the normal ultra- structural picture of the hippocampus (Fig. 12).

## Discussion

A severe recurrent illness called hepatic encephalopathy (HE) is brought on by acute liver failure (ALF). HE includes wide range of neuropsychiatric disorders , from mild cognitive impairment to significant disorientation, motor in coordination, confusion, and coma^27^. TAA has been widely used to induce HE in experimental animals. The pathophysiology of HE may be due to oxidative stress, inflammation and hyper-ammonia^28^. Many studies have focused on identifying therapeutic interventions that can serve to decrease the burden of debilitating neural symptoms in patients with acute liver diseases. Evidence gathered over the last two decades suggests that nutritional supplementation with medicinal herbs or their bioactive compounds such as herbacetin may be used as a protective element against HE because of their strong anti-inflammatory and antioxidant properties^29^.

The hepatoprotective and neuroprotective effects of herbacetin against HE were examined in the current study. We carried out an in vivo experiment using TAA to induce HE in order to clarify the mechanism of herbacetin in alleviating HE. The results of this study show for the first time that herbacetin has neuroprotective properties against HE-related brain damage, via activation of the SIRT1/AMPK signalling pathway. Numerous studies have demonstrated that herbacetin has a variety of benefits that could be linked to its anti-inflammatory and antioxidant properties^30^.

It was documented that TAA-intoxication could alter neurotransmission and affect motor coordination and spatial learning^31^. In the present study, rats administrated TAA showed decreased motor activity, coordination and also decline in learning indices, such as SAP % and novel object recognition which were parallel with similar studies^32^, which showed that experimental rats received TAA shown a decline in motor skill performance, leading to motor and learning impairment due to neuro-inflammation coupled with a large increase in ammonia levels^33^.

Wider researches are needed to determine the precise mechanisms driving HE-induced a decrease in locomotor activity. However; earlier study has highlighted the central role of post-hyper-ammonia metabolic alterations, including altered glutamate/glutamine metabolism, elevated neurosteroids, and dysregulated GABAergic neurotransmission. Increased extracellular glutamate causes the N-methyl d-aspartate (NMDA) type of glutamate receptor to become hyperactive, which causes mitochondrial dysfunction, an excess of ROS, and stimulation of calcium-dependent enzymes, all of which contribute to neurodegeneration and death^34^.

The above-mentioned behavioral tests were significantly improved after receiving herbacetin 20, or herbacetin-40, compared to the TAA group. It's interesting to note that compared to herbacetin 20mg/kg, herbacetin 40mg/kg was better able to repair the cognitive and motor capacities of TAA-intoxicated rats and regain their predicted values. These results show that herbacetin dramatically increase locomotor activity, reduced cognitive deficit, and restored non-spatial orientation learning capacity. Herbacetin might be a promising protective agent for cognitive improvement in HE. Our findings are in line with earlier research which found that Berberine improves spatial learning and memory in TAA-induced liver cirrhosis by improving the blood brain function, oxidative stress and neuro-inflammation^35^.

In the present study, HE rats exhibited notable rises in serum liver enzymes (ALT, AST and ALP) commonly used markers for liver dysfunction. Significant increase in blood levels of ALT, AST and ALP, which denotes a loss of membrane integrity and consequent leakiness in hepatocytes. Another study illustrated elevated levels of these markers after TAA administration and HE induction^36^. Nevertheless, rats treated with herbacetin demonstrated lowered serum levels of ALT, AST, and ALP. Furthermore, herbacetin was found to provide protective effects against acute liver failure.

The current study demonstrated significant rise in blood and brain ammonia levels in the TAA group. One of the primary contributing elements in the etiology of HE is hyper-ammonia Ammonia is a neurotoxic that causes neuro-inflammation, cerebral edema, and oxidative stress^37^. The liver is primarily responsible for metabolizing ammonia under physiological conditions. The hepatic clearance of ammonia is inadequate in HE due to a decrease in hepatic GS that plays a crucical role in ammonia homeostasis, which raises systemic ammonia levels^38^. GS, which is primarily abundant in astrocytes, then allows ammonia to pass through the blood–brain barrier to be detoxified in the brain. As a result, glutamine builds up in the astrocytes, leading to cerebral edema and the progression of HE^39^. These result were in consistent with past researches^40,41^. Another main factor plays a role in pathogenesis of HE is oxidative damage. Additionally, in this study, hepatic encephalopathy (HE) induced by TAA notably elevated brain tissue MDA levels and reduced GSH levels in HE rats, potentially due to increased oxidative stress. Previous studies have demonstrated that TAA administration can lead to liver failure, with potential implications for brain damage. Furthermore, research suggests that using TAA to induce experimental HE can trigger the generation of free radicals and oxidative stress^2^. The present study revealed that herbecetin reduces lipid peroxidation (MDA) and increases GSH levels in brain tissues.

Based on the preceding findings, two pivotal factors for the onset and progression of hepatic encephalopathy (HE) are inflammation and oxidative stress. Numerous studies have shown that hepatic inflammation often manifests within hours of TAA injection. Hence, it is evident that the correction of oxidative damage and hepatic inflammation is integral to mitigating liver damage^42^. According to certain reports, peripheral inflammation, particularly elevated TNF-α and IL-1β expression, might cause neuroinflammation. This encourages the influx of blood cells, particularly lymphocytes, into the brain, which causes the cognitive impairments and motor incoordination^41^. In this study, herbecetin notably lowered the levels of TNF-α and IL-1B in rats, suggesting that its anti-inflammatory effects could be a potential strategy against TAA-induced hepatic encephalopathy.

Moreover, the key metabolic energy sensor; AMPK regulates metabolic homeostasis by controlling various homeostatic mechanisms, such as autophagy and protein degradation, and may detect energy deficit as an elevated AMP/ATP ratio^43^. It has been established that SIRT1 requires AMPK activity. For AMPK phosphorylation, SIRT1 serves as a direct substrate^44^. The available studies have demonstrated that TAA is a potent inducer of neuronal death. The current work additionally showed that TAA-induced neuronal death was linked to AMPK/SIRT1 suppression.

Our findings suggested that the protective impact of herbacetin on autophagy in TAA rats is predominantly mediated by the AMPK/SIRT1 pathway because it was able to restore SIRT1's down regulated expression produced by TAA. In parallel with our study, Dong et al.^45^ noticed that SIRT1 can reduce oxidative stress in the brains of mice. .Moreover, AMPK inhibits the expression of NF-B, IL-1, and TNF- while simultaneously promoting the expression of SIRT1. Reference^46^ and this observation aligns with the outcomes of the current study. SO, herbecetin's has anti-apoptotic, anti-inflammatory, and anti-oxidative effects were most likely caused by its regulation of the AMPK/SIRT1 signaling pathway. In parallel to biochemical and neurological assessments, the hepato- and neurotoxic efects of TAA were further confirmed by histopathological alterations recorded in the hippocampus of the brain. The recorded lesions were showing marked disorganization, marked apoptosis of large pyramidal cells, appear of few vacuoles in Molecular layer and decreased population of granular cells and necrosis.

## Conclusion

The combined findings offer strong proof that herbacetin has neuroprotective effects against HE. Herbacetin preserved liver function and guarded against hyperammonemia-induced neurodegenerative effects, in part via activating SIRT1/AMPK signaling, to successfully mitigate the degenerative effects that accompanied TAA-induced HE. This was demonstrated by the results, which revealed a notable improvement in the cognitive and motor deficiencies, as well as biochemical and molecular markers supported by the results of the histopathological and electron microscope studies.

## Data Availability

The datasets generated during and or analysed during the current study are available from corresponding author on reasonable request.

## Abbreviations

ALI: Acute liver injury
(AnnV): Annexin V
AST: Aspartate aminotranferase
ALT: Alanine aminotranferase
ALK: Alkaline phosphatase
AMPK: AMP-activated kinase
ELISA: Enzyme linked immunosornant assay
DI: Discriminating index
GS: Glutamine synthetase
GSH: Reduced glutathione
H&E: Hematoxylin and eosin
HE: Hepatic encephalopathy
IL-1β: Interleukin 1 B
MDA: Malondialdeyde
NOR: Novel object recognition
ROS: Reactive oxygen species
SD: Standared deviation
TNF-α: Tumor necrosis factor- alpha
SIRT1: Sirtuin 1
TAA: Thioacetamide

## References

[1] Wang W, Wu L, Li Q, Zhang Z, Xu L, Lin C (2018). Madecassoside prevents acute liver failure in LPS/D-GalN-induced mice by inhibiting p38/NF-κB and activating Nrf2/HO-1 signaling. Biomed. Pharmacother..

[2] Hajipour S, Farbood Y, Dianat M, Rashno M, Khorsandi LS, Sarkaki A (2021). Thymoquinone improves behavioral and biochemical deficits in hepatic encephalopathy induced by thioacetamide in rats. Neurosci. Lett..

[3] Sun X, Lv Y, Huang L, Gao H, Ren C, Li J (2020). Pro-inflammatory cytokines serve as communicating molecules between the liver and brain for hepatic encephalopathy pathogenesis and *Lycium*
*barbarum* polysaccharides protection. J. Ethnopharmacol..

[4] Ati H, Fawzy G, El-Gamal AA, Taha A (2015). Phytochemical and biological evaluation of Buddleja polystachya growing in Saudi Arabia. Pak. J. Pharm. Sci..

[5] Li L, Sapkota M, Kim SW, Soh Y (2016). Herbacetin inhibits RANKL-mediated osteoclastogenesis in vitro and prevents inflammatory bone loss in vivo. Eur. J. Pharmacol..

[6] Veeramani C, Alsaif MA, Al-Numair KS (2018). Herbacetin, a flaxseed flavonoid, ameliorates high percent dietary fat induced insulin resistance and lipid accumulation through the regulation of hepatic lipid metabolizing and lipid-regulating enzymes. Chem. Biol. Interact..

[7] Li X (2013). SIRT1 and energy metabolism. Acta Biochim. Biophys. Sin..

[8] Afra HS, Zangooei M, Meshkani R, Ghahremani MH, Ilbeigi D (2019). Hesperetin is a potent bioactivator that activates SIRT1-AMPK signaling pathway in HepG2 cells. J. Physiol. Biochem..

[9] Milner J (2009). Cellular regulation of SIRT1. Curr. Pharm. Des..

[10] Chao LCTP (2012). SIRT1 regulation—It ain’t all NAD. Mol. Cell.

[11] Wang L-F (2017). Inhibition of NAMPT aggravates high fat diet-induced hepatic steatosis in mice through regulating Sirt1/AMPKα/SREBP1 signaling pathway. Lipids Health Dis..

[12] Hou X, Xu S, Maitland-toolan KA, Sato K, Jiang B, Ido Y (2008). SIRT1 regulates hepatocyte lipid metabolism through activating AMP-activated protein kinase. J. Biol. Chem..

[13] Hosseini S (2020). Effect of resveratrol on thioacetamide-induced liver damage in rat models. Hepat. Mon..

[14] Ijaz MU, Mustafa S, Batool R, Naz H, Ahmed H, Anwar H (2022). Ameliorative effect of herbacetin against cyclophosphamide-induced nephrotoxicity in rats via attenuation of oxidative stress, inflammation, apoptosis and mitochondrial dysfunction. Hum. Exp. Toxicol..

[15] Baraka SM, Mowaad NA, Ibrahim S, Korany RMS, El-Sayed AF, Hassan AA (2023). Green synthesized cerium oxide nanoparticles ameliorate hepatic and cognitive dysfunctions in thioacetamide-induced hepatic encephalopathy in rats: Modulation of TLR-4/NF-κB/Caspase-3 signaling pathways. J. Drug Deliv. Sci. Technol..

[16] Sedik AA, Hassan A, Saleh DO (2023). Neuromodulatory role of L-arginine: Nitric oxide precursor against thioacetamide-induced-hepatic encephalopathy in rats via downregulation of NF-κB-mediated apoptosis. Environ. Sci. Pollut. Res..

[17] Khalifa M, Fayed RH, Sedik AA, Khalil HM (2023). Dose-dependent toxic effects of di-(2-ethylhexyl) phthalate in male rats: Focus on behavioral alterations and inducing TLR4/NF-κB signaling pathway. Toxicol. Appl. Pharmacol..

[18] Yu M (2011). Nuclear factor p65 interacts with Keap1 to repress the Nrf2-ARE pathway. Cell Signal..

[19] Konitzer KVS (1963). Direct determination of ammonium in blood and tissue extracts by means of the phenol by chlorite reaction. Clin. Chim. Acta Int. J. Clin. Chem..

[20] Matsumiya HHH (2003). Selective determination of beryllium (II) ion at Picomole per Decimeter cubed levels by kinetic differentiation mode reversed-phase high-performance liquid chromatography with Fluorometric detection using 2-(2 ‘-Hydroxyphenyl)-10- hydroxybenzo [h] quinoli. Anal. Chem..

[21] Vaziri N, Wang X, Oveisi F, Song BJ (2000). Induction of oxidative stress by glutathione depletion causes severe hypertension in normal rats. Hypertens. Free Radic. Biol..

[22] Sun J, Li F, Chen XJ (2004). Effect of ketamine on NF-kappa B activity and TNF-alpha production in endotoxin-treated rats. Ann. Clin. Lab. Sci..

[23] Govindan R, DeVita V (2009). DeVita, Hellman, and Rosenberg’s Cancer: Principles & Practice of Oncology Review.

[24] Sedik AA, Salama M, Fathy K, Salama A (2023). Cold plasma approach fortifies the topical application of thymoquinone intended for wound healing via up-regulating the levels of TGF-ß, VEGF, and α-SMA in rats. Int. Immunopharmacol..

[25] Carleton H, Drury R, Wallington E, Carleton HM, Drury RAB, Wallington EA (1980). Carleton’s Histological Technique.

[26] Reynolds ES (1963). The use of lead citrate of high pH as an electron opaque stain in electron microscopy. J. Cell Biol..

[27] Rose CF, Amodio P, Bajaj JS, Dhiman RK, Montagnese S, Taylor-Robinson SD (2020). Hepatic encephalopathy: Novel insights into classification, pathophysiology and therapy. J. Hepatol..

[28] Lima LCD (2019). Hepatic encephalopathy: Lessons from preclinical studies. World J. Hepatol..

[29] Ijaz MU, Mustafa S, Batool R, Naz H, Ahmed H, Anwar H (2022). Ameliorative effect of herbacetin against cyclophosphamide-induced nephrotoxicity in rats via attenuation of oxidative stress, inflammation, apoptosis and mitochondrial dysfunction. Hum. Exp. Toxicol..

[30] Li M, Qi Z, Hao Y, Lv C, Jia L, Wang J (2017). New adducts of iriflophene and flavonoids isolated from sedum aizoon l. with potential antitumor activity. Molecules.

[31] Dadsetan S, Balzano T, Forteza J, Cabrera-Pastor A, Taoro-Gonzalez L, Hernandez-Rabaza V (2016). Reducing peripheral inflammation with infliximab reduces neuroinflammation and improves cognition in rats with hepatic encephalopathy. Front. Mol. Neurosci..

[32] Leke R, de Oliveira DL, Mussulini BHM, Pereira MS, Kazlauckas V, Mazzini G (2007). Impairment of the organization of locomotor and exploratory behaviors in bile duct-ligated rats. PLoS One.

[33] Méndez M, Méndez-López M, López L, Aller MÁ, Árias J, Cimadevilla JM (2008). Spatial memory alterations in three models of hepatic encephalopathy. Behav. Brain Res..

[34] Cooper AJL, Jeitner TM (2016). Central role of glutamate metabolism in the maintenance of nitrogen homeostasis in normal and hyperammonemic brain. Biomolecules.

[35] Hajipour S, Farbood Y, Dianat M (2023). Effect of berberine against cognitive deficits in rat model of thioacetamide-induced liver cirrhosis and hepatic encephalopathy (behavioral, biochemical, molecular and histological evaluations). Brain Sci..

[36] Hajovsky H, Hu G, Koen Y, Sarma D, Cui W, Moore DS (2012). Metabolism and toxicity of thioacetamide and thioacetamide S-oxide in rat hepatocytes. Chem. Res. Toxicol..

[37] El-Marasy SA, El Awdan SA, Abd-Elsalam RM (2019). Protective role of chrysin on thioacetamide-induced hepatic encephalopathy in rats. Chem. Biol. Interact..

[38] Chepkova AN, Sergeeva OA, Görg B, Haas HL, Klöcker N, Häussinger D (2017). Impaired novelty acquisition and synaptic plasticity in congenital hyperammonemia caused by hepatic glutamine synthetase deficiency. Sci. Rep..

[39] Qvartskhava N, Lang PA, Görg B, Pozdeev VI, Ortiz MP, Lang KS (2015). Hyperammonemia in gene-targeted mice lacking functional hepatic glutamine synthetase. Proc. Natl. Acad. Sci. U. S. A..

[40] Khodir AE, Said E (2020). Nifuroxazide attenuates experimentally-induced hepatic encephalopathy and the associated hyperammonemia and cJNK/caspase-8/TRAIL activation in rats. Life Sci..

[41] Farjam M, Dehdab P, Abbassnia F, Mehrabani D, Tanideh N, Pakbaz S (2012). Thioacetamide-induced acute hepatic encephalopathy in rat: Behavioral, biochemical and histological changes. Iran Red Crescent Med. J..

[42] Khodir AE, Said E (2020). Nifuroxazide attenuates experimentally-induced hepatic encephalopathy and the associated hyperammonemia and cJNK/caspase-8/TRAIL activation in rats. Life Sci..

[43] Lau AW, Liu P, Inuzuka H, Gao D (2014). SIRT1 phosphorylation by AMP-activated protein kinase regulates p53 acetylation. Am. J. Cancer Res..

[44] Price LN, Gomes PA, Ling JYA (2012). SIRT1 is required for AMPK activation and the beneficial effects of resveratrol on mitochondrial function. Cell Metab..

[45] Dong YT, Cao K, Tan LC, Wang XL, Qi XL, Xiao Y (2018). Stimulation of SIRT1 attenuates the level of oxidative stress in the brains of APP/PS1 Double transgenic mice and in primary neurons exposed to oligomers of the amyloid-β peptide. J. Alzheimers Dis..

[46] Yuan T, Yang T, Chen H, Fu D, Hu Y, Wang J (2019). New insights into oxidative stress and inflammation during diabetes mellitus-accelerated atherosclerosis. Redox Biol..

